# Cancer Metabostemness and Metabolic Reprogramming via P2X7 Receptor

**DOI:** 10.3390/cells10071782

**Published:** 2021-07-14

**Authors:** Izadora Lorrany Alves Rabelo, Vanessa Fernandes Arnaud-Sampaio, Elena Adinolfi, Henning Ulrich, Claudiana Lameu

**Affiliations:** 1 Departamento de Bioquímica, Instituto de Química, Universidade de São Paulo, São Paulo 05508-000, Brazil; ilorrany@usp.br (I.L.A.R.); vanessa.arnaud@usp.br (V.F.A.-S.); henning@iq.usp.br (H.U.); 2 Department of Medical Sciences, Section of Experimental Medicine, University of Ferrara, 44121 Ferrara, Italy; elena.adinolfi@unife.it

**Keywords:** cancer stem cells, stemness, P2X7 receptor, P2X7A, P2X7B, purinergic signaling, metastasis, metabolism, metabolic reprogramming, chemotherapy and chemoresistance

## Abstract

The heterogeneity of tumor cell mass and the plasticity of cancer cell phenotypes in solid tumors allow for the insurgence of resistant and metastatic cells, responsible for cancer patients’ clinical management’s main challenges. Among several factors that are responsible for increased cancer aggression, metabolic reprogramming is recently emerging as an ultimate cancer hallmark, as it is central for cancer cell survival and self-renewal, metastasis and chemoresistance. The P2X7 receptor, whose expression is upregulated in many solid and hematological malignancies, is also emerging as a good candidate in cancer metabolic reprogramming and the regulation of stem cell proliferation and differentiation. Metabostemness refers to the metabolic reprogramming of cancer cells toward less differentiated (CSCs) cellular states, and we believe that there is a strong correlation between metabostemness and P2X7 receptor functions in oncogenic processes. Here, we summarize important aspects of P2X7 receptor functions in normal and tumor tissues as well as essential aspects of its structure, regulation, pharmacology and its clinical use. Finally, we review current knowledge implicating P2X7 receptor functions in cancer-related molecular pathways, in metabolic reprogramming and in metabostemness.

## 1. Introduction

P2X7 receptor is an ATP-gated purinergic ion channel widely present in different cells and tissues, such as stem cells [1], brain [2], intestine [3], kidney [4] and mostly expressed in immune [5] and several cancer cells [6,7,8].

Overstimulation of the receptor can activate a large pore in the membrane. This is not an exclusive feature of P2X7 receptor, as it has also been identified in TRPV_1_, ASICs, P2X2 and P2X4 receptors [9]. Nonetheless, while further receptors open pore upon prolonged stimulation, the P2X7 receptor takes only milliseconds to do the same [9]. The pore opening is responsible for many of its functions, including tumor cell death [10]. In contrast, its tonic activation promotes tumor growth [11]. It has been confirmed that the P2X7 receptor exerts these dual activities in the majority of cancers.

The P2X7 receptor binds its physiological agonist ATP with low affinity. Therefore, its activation is only achieved when the level of ATP is significantly elevated in the extracellular space [7]. Since, ATP concentration is extremely higher in tumor microenvironment compared to healthy tissues, the P2X7 receptor may function as key signal transducer in the communication of TME and cancer cells [12]. In this way, blockade of the P2X7 receptor emerges as a potential effective [9] anticancer treatment.

Although the role of the P2X7 receptor in oncogenesis has not been fully elucidated, the link between functions attributed to this receptor and tumor cells has been broadly recognized [13]. P2X7 receptor stimulation can contribute to tumor biology in different ways, such as maintenance of cancer stem cells (CSC), tumor progression, chemoresistance and metastasis [14].

The emerging cancer hallmark metabolic reprogramming is a fundamental aspect of cancer metastasis and therapy resistance [15]. P2X7 receptor expression has a pivotal role in metabolic diseases and cancer metabolic reprogramming [16]. The receptor tonic stimulation increases mitochondrial potential (Δψm), the glycolytic rate, glycogen storage and oxidative phosphorylation efficiency. On the other hand, its overstimulation is detrimental for cells, resulting in decreased Δψm, mitochondrial fragmentation and cell death [17,18,19,20]. Besides, the absence of the P2X7 receptor reduces mRNA and protein expression of fatty acid metabolism key enzymes, such as FASN and acetyl-CoA carboxylase (ACC), and increases serum triglyceride and cholesterol levels, glucose intolerance and insulin resistance, which decreases stemness, proliferation, survival, invasiveness and therapeutic resistance [21]. This evidences strongly correlate the involvement of the receptor in metabostemness and metabolic reprogramming of cancer cells.

Metabostemness may be referred to as "the metabolic parameters causally controlling or functionally substituting the epitranscriptional orchestration of the genetic reprograming that redirects normal and tumor cells toward less differentiated (CSCs) cellular states" [22]. Here, we summarize important aspects of P2X7 receptor structure, regulation, pharmacology and its clinical use. Moreover, the recent findings connect the P2X7 receptor actions with metabostemness and with metabolic reprogramming of cancer cells.

## 2. The Purinergic Signaling System—Structural Insights Focusing on the P2X7 Receptor

Purinergic or pyrimidinergic nucleotides and nucleosides present in the extracellular space act as co-transmitters, neuromodulators or ligands in cell signaling events, thus, participating in purinergic signaling [23,24]. The purinergic system is composed of P1, P2 and recently proposed, P0 receptors (P1Rs, P2Rs and P0Rs) [25]. P1Rs are G protein-coupled receptors responsive to adenosine. P2Rs are either ionotropic channels responsive to ATP (P2XRs) or metabotropic/G protein-coupled receptors responsive to ATP, ADP, UTP, UDP or UDP-glucose (P2YRs). Finally, P0Rs are G protein-coupled receptors responsive to adenines [26].

Seven types of P2X subunits are known so far, ranging from P2X1 to P2X7. Each P2XR ion-channel is composed of three subunits, either in homo- or heterotrimeric compositions [27]. Each subunit contains two hydrophobic transmembrane domains (TM1 and TM2) linked by a large, glycosylated and cysteine-rich extracellular loop [28,29,30]. In the intracellular space, the P2X subunits present a short *N*-terminal domain with approximately 30 amino acid residues [31] and a length-variable C-terminal domain [30] with 30–240 amino acid residues [31]. TM domains participate in channel gating and conductance, and may be involved in the differential desensitization kinetics of P2XR subtypes [31,32]. TM1 is associated chiefly with channel opening and receptor sensitization [33], whereas TM2 internally coats the pore permeable to sodium, potassium and calcium [24,34]. P2XR-intracellular domains have been strongly related to membrane trafficking, homologous desensitization, protein-protein interactions and phospholipids modulation [28,31,35,36,37]. The P2X7 receptor almost does not desensitize, and sustained ATP stimulation leads to more distinct responses than acute stimulation does [31,32]. 

The P2XR ectodomain presents three available ATP-binding sites, although it was recently showed that the occupancy of two is enough to activate these receptors [38,39]. The sequential binding of each ATP molecule leads to asymmetrical conformational changes that decrease agonist affinity to the binding site, leading to a negative cooperativity mechanism [40]. Regardless of amino acid residues underlying ATP-binding being highly conserved among different subtypes [31], the P2X7 receptor has the lowest ATP sensitivity within the P2XR family [37].

As response to ATP stimulation, the P2X7 receptor channel opening promotes mono- or divalent cation currents, generally by Na^+^ and Ca^2+^ influx and K^+^ efflux, leading to cell depolarization and downstream Ca^2+^ signaling events. Sustained stimulation drives a non-selective long-lasting opening, which allows the permeation of larger molecules of up to 900 Da, including common fluorescent dyes [10,41].

There are several isoforms of the P2X7 receptor, named P2X7A-J. The P2X7 receptor can be assembled by just one of them or by a composition of two or three different isoforms [42]. P2X7 receptor-related functions in physiological or oncological processes are associated with the expression levels of its genetic variants [43,44]. Among them, P2X7A and B isoforms are the most studied in humans. P2X7A has a C-terminal crucial for opening membrane large pores causing apoptosis, while P2X7B has a reduced C-terminal, being incapable of inducing cell death [11], while it is able to promote proliferation of stem and tumor cells [43,44] and participates in the cell differentiation process [44]. Although the role of isoforms for cancer metabolism reprogramming and metabostemness has been clarified yet, we hypothesize that a differential contribution of these isoforms account for cancer cell survival and self-renewal, metastasis and chemoresistance. Understanding the functions of P2X7 isoforms on cancer metabolism may open avenues to more efficient therapy against chemoresistant and metastatic tumor.

## 3. Metabolic Pathways Driving Cancer Cell Survival and Stemness

Metabolic reprogramming is recognized as a cancer hallmark [45], being interpreted both as a driver of malignant transformation and a consequence of this transformation. Current hypotheses are based on the assumption that metabolic alterations happen due to increased energy demands of cancer cells or decreased availability of nutrients or oxygen. Increased metabolism would be a mere consequence of cancer transformation. On the other hand, metabolism may be an active player in determining cell fate, such as malignancy progression. Indeed, it is true that metabolic changes confer a survival advantage when a tumor is already established [15].

It is generally thought that cancer cells make little use of mitochondria and mainly depend on anaerobic glycolysis for their energy demand even in presence of oxygen, a phenomenon called Warburg effect [46]. Nowadays, it is known that uncontrolled mitochondria bioenergetics has an important role in cancer metabolism and tumorigenesis [47], i.e., through the generation of ROS following disruption of regular mitochondrial homeostasis [48], strongly driving cancer progression [49].

Progressively several studies have been shedding light on the roles of mitochondria in the stemness state of cancer cells as well as on their contributions to metastasis and therapeutic resistance, along with strategies to identify and to eradicate these cells [50,51,52] by targeting their mitochondria [53,54].

It is known that Δψm is heterogeneous among cell populations and is related to the level of cell commitment and differentiation. Hippocampal neuroblasts, for instance, develop a significant Δψm increase when induced to differentiate in the presence of retinoic acid treatment. Accordingly, Δψm is also related to tumorigenic properties of stem cells. Among mouse embryonic stem cells, those with higher Δψm displayed augmented tumorigenic potential, although they presented similar levels of surface pluripotency marker expression and similar morphology [55].

Metabolic pathways and the distribution of mitochondria, which is related to the balance between symmetric and asymmetric divisions, tightly control stem cell populations [56]. Further, mitochondrial dynamics are involved in proliferating and quiescent states of cancer stem cells. A large fraction of mitochondria forms a tubular network in proliferating cells, while quiescent CSCs localized in the core of the tumorspheres, a highly hypoxic environment, are characterized by donut-shaped mitochondria [57]. In agreement, mitochondrial tubular morphology was donut-shaped under hypoxia-reoxygenation stress, a protective mitochondrial mechanism to aid adaptation and functional recovery [58].

In the context of metabolic reprogramming, CSCs have a mitochondria-centric energy metabolism, giving them the ability to consume limited available nutrients, such as fatty acids to generate ATP, NADPH, tricarboxylic acid (TCA) cycle intermediates, nucleotide bases, electron acceptors and others, favoring cancer cell survival and proliferation signaling [49] and epigenetic regulation, but also by genetic-independent mechanisms tightly related to metabolic reprogramming [22,59].

In glioblastoma, glucose uptake and lactate output was even more expressive in CSCs than in the tumor bulk, in addition to upregulation of pyruvate dehydrogenase kinase-1 (PDK-1) expression levels [60]. However, lower oxygen and glucose consumption rates, intracellular ATP and ROS levels were observed in lung CSCs compared to differentiated cells, as well as oxidative phosphorylation preference for energy supply [61]. A chemoresistant stem-like side population (SP) within human tumors characterized by Hoechst33342 efflux capability presents higher glycolytic activity in comparison to their efflux-incapable counterparts [62]. Importantly, the proportion of SPs increases when glucose is abundant. This regulation depends on Akt pathway activation due to AMP-activated protein kinase (AMPK) suppression by increased intracellular ATP concentration [62].

Fatty acid (FA) metabolism includes anabolic and catabolic pathways essential to structure and sustain the cellular membrane, to supply energy and to produce intermediates mediating several signalling pathways. The fine-tuning balance between FA synthesis and oxidation can be easily perturbed by aberrant expression of the genes involved in these processes. Once this occurs, inadequate FA levels induce lipid accumulation and the general phenotypes of malignant cancers appear, being also strongly correlated with the presence of CSCs within the tumor cell population, as well as resistance to therapy [59].

The overexpression of lipogenic enzymes, such as fatty acid synthase (FASN) in several cancers has been correlated with cancer progression, poor prognosis and resistance to chemotherapy [63,64]. FASN is the enzyme whereby the condensation between acetyl-CoA and malonyl-CoA, at the final catalytic step of FA synthesis, produces palmitate, and has been indicated as an emerging target to cancer [65].

Glioblastoma stem cells expressing stem cell markers happen to be the same cells presenting upregulation of FASN. Interestingly, when the enzyme is inhibited by cerulenin, not only expression levels of stem cell markers, as well as the numbers of tumorspheres, GSCs proliferation and invasiveness are diminished [66].

## 4. P2X7 Receptor Relevance in Metabolism

The P2X7 receptor participates in regulation of mitochondrial functions, and the tone of ATP stimulation is crucial for determining downstream events. While tonic stimulation has a stabilizing effect on mitochondrial homeostasis, resulting in increased mitochondrial potential (Δψm) and oxidative phosphorylation efficiency; overstimulation has a killing effect, resulting in decreased Δψm, mitochondrial fragmentation and cell death [17,18]. Therefore, the expected effect of a tonic eATP (extracellular ATP) stimulation of P2X7 receptor in the tumor microenvironment is a combination of both increased glycolytic rate and oxidative phosphorylation efficiency, which contributes to ATP synthesis and anabolic responses [19].

Also, P2X7 receptor-induced effects on Δψm may play a role in determining stem cell fate, as well as tumorigenic potential. Indeed, Δψm is reduced in several cell types lacking P2X receptors, including HEK293 cells, human and mouse embryonal fibroblasts and microglia [17,18,67].

Although the role of P2X7 receptor in the balance between symmetric and asymmetric divisions is not yet clear, many pieces of evidence showed that P2X7 receptor stimulates the maintenance of cancer stem cells [14,67] and possibly asymmetric cell division [68]. An increase in ATP release and pericellular concentration was related to greater mitochondria numbers and activity in leukemia cells, resulting in cancer cell proliferation [69]. Importantly, P2X7 receptor-mediated effects on mitochondria morphology were reported following cell death or pseudoapoptosis induction [70]. However, respective functional implications need yet to be elucidated.

The downstream effects of P2X7 receptor activation culminate in metabolic changes, with some of them resembling the Warburg effect. P2X7 receptor expression in HEK-293 cells induced a metabolic reprogramming, which favored the adaptation of these cells to adverse conditions, such as growth in serum-starved [71] and low glucose media [72]. In addition to that, the presence of the receptor resulted in increased expression of glycolytic enzymes, while inhibiting pyruvate dehydrogenase and enhancing lactate production [72]. Such relevant findings where not only obtained with HEK-293 cells, but also similarly verified in a human neuroblastoma cell line [72].

P2X7 receptor expression was also associated with the rise of glycogen storage probably through down-regulation of glycogen synthase kinase 3β (GSK3β) activity [20].

**Figure 1:**
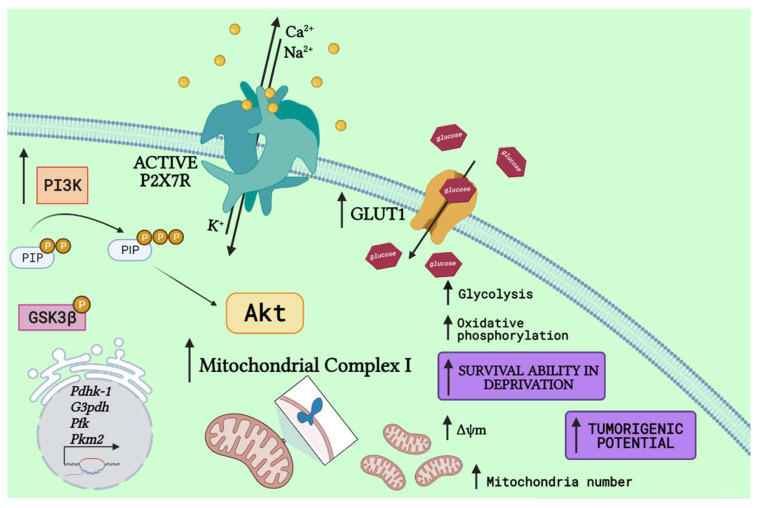
P2X7 receptor activation drives a metabolic shift. P2X7 receptor (P2X7R) activation drives PI3K activity, generating phosphatidylinositol (3,4,5)-trisphosphate (PIP3) and ultimately enhancing Akt signaling. Following, it drives upregulation of glycolytic-related enzymes, such as pyruvate dehydrogenase kinase-1 (PDHK-1) [72], a similar response to that observed in stem cells in the presence of glucose [62], and others, as glyceraldehyde 3-phosphate dehydrogenase (G3PDH), phosphofructokinase (PFK), and pyruvate kinase M2 (PKM2) [72]. Despite glycolytic improvements, the P2X7R is also related to increased expression of glucose transporters (GLUT) [72,73], increasing glucose uptake and oxidative phosphorylation, matched to higher expression of mitochondrial complex 1 and potential (Δψm), as well as mitochondria number [18] and increased glycogen storages through GSK3β phosphorylation [20]. Altogether, these metabolic shifts make cells highly capable of surviving in deprivation conditions and enhance the tumorigenic potential of cancer cells (Created with BioRender.com).

P2X7 receptor stimulation in neuroblastoma cells increases intracellular ATP content [71] and enhances the PI3K/Akt pathway [20], suggesting a possible role in metabolic regulation of stem-like populations. In addition, glucose induces expression of glycolysis-related proteins, such as hexokinase-1 (HK-1) and pyruvate dehydrogenase kinase (PDK)-1 in CSCs, due to activation of the Akt pathway [62]. Interestingly, upregulation of a very similar set of proteins was verified upon P2X7 receptor stimulation in neuroblastoma cells, including PDK-1 [72], as shown in Figure 1.

The P2X7 receptor has been related to FA metabolism in a way that P2X7KO mice presented reduction at mRNA and protein expression level of key enzymes, such as FASN and acetyl-CoA carboxylase (ACC). Besides, the absence of P2X7 receptor did not only result in higher serum triglyceride and cholesterol levels, but also in glucose intolerance and insulin resistance [21].

**Figure 2:**
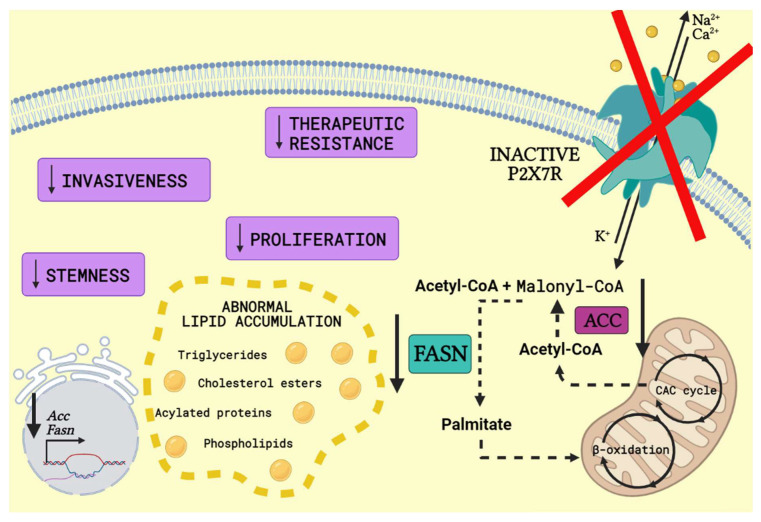
P2X7 receptor knockout or antagonism in FA metabolism. P2X7 receptor (P2X7R) knockout or antagonism reduces mRNA and protein expression level of fatty acid metabolism key enzymes such as acetyl-CoA carboxylase (ACC) and fatty acid synthase (FASN). ACC converts acetyl-CoA generated from citric acid cycle (CAC cycle) into malonyl-CoA. Following, FASN catalyses the conversion of acetyl-CoA and malonyl-CoA into palmitate, which enters mitochondria and goes through β-oxidation, producing long-chain saturated fatty acids. The knockout or antagonism of P2X7R surprisingly results in abnormal lipid accumulation, such as higher serum triglyceride and cholesterol levels, which is associated with hepatic steatosis [75]. Moreover, FASN inhibition is related to a decrease in the number of cancer stem cells, proliferation, invasiveness and resistance to therapy (Created with BioRender.com).

Metabolic in vivo correlation with P2X7 receptor was observed, as body weight gain, abnormal lipid accumulation, adipocyte hyperplasia, increased fat mass and ectopic distribution was caused by P2X7 receptor loss of function in P2X7KO mice. Dysregulated energy homeostasis favored fatty acid oxidation not only in P2X7KO mice but also in WT mice treated with the selective P2X7 receptor antagonist A804598 [74], as seen in Figure 2.

The same group stimulated P2X7 receptor in vivo with its agonist BzATP, which increased metabolic rate and O_2_ consumption and decreased respiratory rate and upregulated NADPH oxidase 2 in gastrocnemius and tibialis anterior muscles [76]. These observations correlate P2X7 receptor activity with fatty acid catabolic pathway upregulation and cancer cell plasticity.

Malignant hematopoietic stem cells have also been shown to overexpress P2X7 receptors, if compared to their non-pathological counterpart [77,78]. In particular, acute myeloid leukemia (AML)-associated leukemic initiating cells (LICs) formed, proliferated, renewed and homed at the endosteal niche in a P2X7 receptor dependent fashion [79]. Interestingly, P2X7 receptor upregulated LIC growth and self-renewal via increased activity of phosphoglycerate dehydrogenase (PHGDH), a central enzyme in glycine and serine metabolism. Other key proteins of signaling pathways, which were in their expression levels upregulated in AML by P2X7 receptors, include cAMP response element-binding protein (CREB) [79], Pre-B cell leukemia transcription factor 3 (Pbx3) [80] and c-myc [81].

## 5. Anti-P2X7 Receptor Drugs in Effectivity Studies or in Use for Cancer Therapy

The P2X7 receptor has become a target for anti-tumor therapy with promising outcomes in various tumors models [7]. Antagonists have been synthesized and described for in vitro and in vivo use to revert P2X7 receptor-mediated effects on the progression of cancer and non-cancer diseases. ATP analogues, such as periodate-oxidized ATP (oATP), have been used for P2X7 receptor antagonism in vivo [82]. However, this compound is not specific, as it also inhibits other P2 receptor subtypes. Brilliant Blue-G (BBG), a non-competitive antagonist of the P2X7 receptor, showed its biological activity by reverting loss of dopaminergic neurons in an animal model of Parkinson’s disease [83] and blocking P2X7 receptor-promoted tumor engraftment and metastasis [6]. Although the BBG is approved for human use as food dye, this molecule is also not selective for P2X7 receptors, also inhibiting P2X4 and P2X1 receptors, besides affecting other proteins, including sodium channels [84] and pannexin-1 [85]. Another important factor is the limited systemic therapeutic use of BBG due to its blue staining properties of the retina. Formulations that improve its solubility and bioavailability might make BBG a more attractive molecule for in vivo applications.

Some P2X7 receptor antagonists, such as the allosteric-acting compounds A-740003 and AZ10606120, and the competitive-acting A-804598 [86,87] are more selective for the receptor, since they act in nanomolar concentrations. These antagonists were successfully used in in vitro and in vivo preclinical models to reduce P2X7 receptor-dependent cell growth and metastasis [7]. The oncological conditions, in which P2X7 receptor antagonism were efficacious in reducing cancer progression include, but are not limited to, breast cancer [88,89], melanoma [90,91,92], neuroblastoma [20], mesothelioma [8], glioma [93] and AML [79,80,81,92]. Notably, the P2X7 receptor antagonism reduced cancer aggressiveness acting at metabolic pathways in some of these models, such as AML, neuroblastoma and glioma [20,81,83,94].

The potent P2X7 receptor antagonist AZ10606120 significantly reduced proliferation of both U251 and surgically resected human high-grade glioma tumor cells [95]. Moreover, a high-affinity monoclonal antibody (4B3A4 mAb) was designed for blocking the human P2X7 receptor, by binding to its extracellular domain. P2X7 receptor activity was effectively blocked by the 4B3A4 mAb, once Ca^2+^ entry and YO-PRO-1 uptake stimulated by ATP were significantly reduced [94], making this compound promising for cancer therapy.

The CNS-penetrating drugs JNJ-42253432 and JNJ-47965567 were developed for treatment of brain diseases, including epilepsy [96]. Furthermore, P2X7 receptor inhibition has been subject in clinical trials for therapy of a number of inflammation-related disorders, including rheumatoid arthritis and inflammatory bowel disease [97]. Notably, the selective oral-administered AZD9056 P2X7 receptor inhibitor was effective in reducing inflammation in patients suffering from Crohn’s disease in clinical trials [98]. Hopefully, P2X7 receptor antagonists currently tested for clinical safety, i.e., CE-224535 and emodin, will be evaluated in clinical settings for their capabilities in reverting tumor progression.

Non-functional variants of P2X7 receptor (nfP2X7R), which contain variations in the extracellular loop region, are broadly expressed in patient tumor samples and have been proven to be fundamental to cancer cell survival [99]. Antibodies developed to recognize nfP2X7R were called E200, for targeting the amino acid sequence in the range 200–216 [99]. These antibodies are being used as biological drugs for basal cell carcinoma treatment and have already passed phase I clinical trials, shown to be safe and tolerable [100]. Small-molecule antagonists, on the other hand, have resulted in several patents claimed by pharmaceutical companies, but they have not yet reached clinical trials [101]. However, animal model results have been promising [7,102]. An interesting review has gathered important studies and trials for drug development against P2X7 receptor in several diseases [103].

A recent study showed the significant anti-tumor activity of ATP-decorated and doxorubicin-loaded mesoporous silica with bio-mineralization of calcium carbonate against the doxorubicin-resistant highly aggressive and metastatic Dalton’s murine lymphoma. The nanocomposite improved its capability of inducing apoptosis via P2X7 receptor activation, when compared to doxorubicin alone [104]. Doxorubicin and the similar chemotherapeutic daunorubicin are part of the anthracyclines drugs family, which increase extracellular ATP levels in the tumor microenvironment [105,106]. Interestingly, both doxorubicin and daunorubicin cellular uptake and consequent cytotoxicity are facilitated by P2X7 receptor variant A-promoted macropore opening [81,107]. On the other hand, the P2X7 receptor variant B protects cells from daunorubicin toxicity and even stimulates their proliferation, probably due to a daunorubicin-dependent ATP concentration increase in the tumor microenvironment. Consequently, in AML, chemotherapy with daunorubicin upregulated P2X7B receptor and downregulated P2X7A receptor expression, resulting in the overexpression of P2X7B receptors in AML-relapsing patients. In view of relapse and chemoresistance being the leading causes of death by AML, the P2X7B receptor is undoubtedly an attractive therapeutic candidate for this pathology [81].

While various studies in course, such as antibody development and clinical trials targeting the P2X7 receptor for cancer treatment, are promising, almost none of them addresses its isoforms in this big picture involving metabostemness, tumorigenesis and metastasis. However, for effectiveness of drug development, the whole scenario needs to be considered - not only the structure of the target of interest.

## 6. Final Remarks

Cancer is a complex and integrative disease hijacking entire body energy and functioning in order to grow and win. Therefore, interactions between the P2X7 receptor and its isoforms with metabolic regulation mechanisms are needed for successful interruption of the oncogenic process.

Aspects of P2X7 receptor structure and activity modulation by ligands within the extracellular milieu result from fatty acid metabolism. The P2X7 receptor C-terminal domain is implicated in most downstream effects of the receptor, including pore-formation and signal transduction [34], while the *N*-terminal domain may undergo alternative splicing dictating the sensitivity of different immune cells to extracellular NAD^+^ and ATP [108]. Receptor modulation by membrane phosphoinositides (PIPn), anionic signaling phospholipids, happens through indirect interactions with the C-terminal tail [36].

While cholesterol-rich membranes may inhibit P2X7 receptor functions, the presence of lipids, such as sphingomyelin, phosphatidylglycerol, and phosphoinositides (PIPn), specifically PI(4,5)P_2_, may promote its activity. Interestingly, PIPn modulation cross-talks with ubiquitous signaling pathways, such as those initiated by PI(4,5)P_2_ hydrolysis. PI(4,5)P_2_ is hydrolyzed by phospholipase C (PLC), generating inositol-triphosphate (IP_3_) and diacylglycerol (DAG) [36,109].

In addition, the rate of agonist-evoked pore formation by P2X7 receptors can also be regulated by cholesterol-membrane levels. Acute depletion of cholesterol increases the rate of large pore formation, while its presence diminishes it, protecting cells from death [110].

Cellular membrane lipid composition is an important factor for pore formation. A truncated (lacking C-terminal) panda P2X7 receptor variant in controlled lipid composition exosomes stimulated with ATP alone was sufficient to allow a dye-permeable pore opening [109]. In addition, inhibition of the P2X7 receptor was induced by cholesterol interactions with TM2, preventing the receptor-induced large pore formation. Palmitoylation of cysteine residues in the C-terminal domain also prevents this interaction, protecting TM2 and allowing pore opening. In cholesterol-rich membranes, when the C-terminal is absent, TM2 remains unprotected and large pore opening is inhibited [109]. Excellent reviews are available to further delve into P2X and P2Y receptor pharmacology [111,112].

Metabolic targets associated with P2X7 receptor modulation, as well as its related cellular events are summarized in Table 1

The current hypothesis based on the assumption that metabolic adjustments occur due to increased energy demands of cancer cells or decreased availability of nutrients or oxygen put the metabolism in a passive position. These studies often do not consider that changes in metabolism may determine cell fate and stemness. Drug resistance must be faced not only as a product of natural selection, but most importantly as a phenomenon linked to cell plasticity that is triggered and reversed by environmental cues driving epigenetic changes. Uncovering how the environment may make cells more sensitive to chemotherapy is a current struggle that must be overcome. Cellular metabolism actively drives tumorigenesis and stemness, not only because of tumor establishment and cancer cells requirements. Recent studies try to understand how the P2X7 receptor modulates the metabolic reprogramming of cancer cells, such as intracellular ATP production, enabling cell division and cytoskeleton changes, which are necessary for tumor progression and metastasis. In addition to that, understanding how the receptor modulates metabostemness of cancer cells is also important. We already know that P2X7 receptor expression favors stemness of embryonic cells [44]. For that reason, together with the knowledge that the two functional P2X7 receptor isoforms may have opposite contributions to cancer biology, we believe the receptor is strongly related to the stemness balance within tumor mass, tumorigenesis, chemoresistance and metastasis [14].

The P2X7 receptor is an important molecule not only in oncogenic processes but also in normal tissues. Several P2X7 receptor antagonists have been synthesized for in vitro and in vivo use to revert P2X7 receptor-induced effects on the progression of cancer and other diseases and some of them underwent clinical trials. However, none of them has connected P2X7 receptor expression and activity modulation with metabostemness and cancer.

Metabolic reprogramming is essential for stemness, cancer metastasis and therapy resistance. Therefore, a solid understanding of P2X7 receptor expression, its regulation and their role in metabolic diseases and cancer metabostemness, as well as metabolic reprogramming are essential to ameliorate current anti-tumoral therapies.

## Figures and Tables

**Table 1:** Summary of metabolic targets modulated by P2X7 receptor expression and/or activation and observed cellular responses.

Metabolic Target	P2X7 Receptor Effect	Related Cellular Events	Available Evidence
Complex I protein	Protein levels: Upregulated on P2X7R-expressing cells [18]	Increased mitochondrial potential, increased respiration	HEK 293 cells overexpressing P2X7R vs. wild-type; N13 microglia cells sufficient vs. deficient for P2X7R
Complex II protein	Protein levels: Downregulated on P2X7R-expressing cells [18]	Not reported	HEK 293 cells overexpressing P2X7R vs. wild-type
GLUT1	Protein levels: Upregulated on P2X7R-expressing cells [72]	Growth in absence of serum or low glucose; increased cellular ATP content	HEK 293 cells overexpressing P2X7R vs. mock-transfected cells; human neuroblastoma cells
GLUT2	Protein levels on cell surface: Downregulated by P2X7R activation [73]	Reduced glucose transport	Pharmacologically activated (BzATP 100 µM) intestinal epithelial cells (IEC)-6 and Caco-2 cells
G3PDH	mRNA expression levels: Upregulated on P2X7R-expressing cells [72]	Growth in absence of serum or low glucose; increased cellular ATP content	HEK 293 cells overexpressing P2X7R vs. mock-transfected cells; human neuroblastoma cells
PFK	Protein levels: Upregulated on P2X7R-expressing cells [72]	Growth in absence of serum or low glucose; increased cellular ATP content	HEK 293 cells overexpressing P2X7R vs. mock-transfected cells, in low glucose conditions; human neuroblastoma cells
PKM2	Protein levels: Upregulated on P2X7R-expressing cells [72]	Growth in absence of serum or low glucose; increased cellular ATP content; increased glycolysis	HEK 293 cells overexpressing P2X7R vs. mock-transfected cells, in low glucose conditions; human neuroblastoma cells
PDHK1	Protein levels: Upregulated on P2X7R-expressing cells [72]	Growth in absence of serum or low glucose; increased cellular ATP content; increased glycolysis	HEK 293 cells overexpressing P2X7R vs. mock-transfected cells, in low glucose conditions; human neuroblastoma cells
PDH	Enzyme activity: Downregulated on P2X7R-expressing cells [72]	Growth in absence of serum or low glucose; increased cellular ATP content; increased glycolysis	HEK 293 cells overexpressing P2X7R vs. mock-transfected cells; human neuroblastoma cells
GSK3β	Phosphorylated protein levels: Upregulated (reduced enzyme activity) on P2X7R-expressing cells/upon P2X7R activation [20]	Increased glycogen stores; tumor cell survival	Human neuroblastoma cells silenced for P2X7 by shRNAs vs. scrambled control; neuroblastoma cells pharmacologically modulated by agonists (BzATP or ATP) or inhibited by AZ10606120 or A740003
NADPH oxidase 2	Protein levels on skeletal muscle: Upregulated upon P2X7R activation [76]	Increased metabolic rate, O_2_ consumption, decreased respiratory rate	P2X7 receptor systemic activation in mice (BzATP, 1 mg/kg)
ACC (acetyl-CoA carboxylase)	mRNA and protein levels: Downregulated in knockout mice [21]	Glucose intolerance, increased serum triglycerides and cholesterol levels	P2X7^−^/^−^ mice vs. wild-type
FASN (fatty acid synthase)	mRNA and protein levels: Downregulated in knockout mice [21]	Glucose intolerance, increased serum triglycerides and cholesterol levels	P2X7^−^/^−^ mice vs. wild-type
PHGDH	mRNA and protein levels: Downregulated in knockdown cells [79]	Lower serine levels; reduced migration, homing and self-renewal abilities	Leukemia initiating cells of mice with MLL-AF9 induced AML (Acute myeloid leukemia); P2X7 knockdown by shRNAs

## Data Availability

Not applicable.
